# Levelized cost estimates of solar photovoltaic electricity in the United Kingdom until 2035

**DOI:** 10.1016/j.patter.2023.100735

**Published:** 2023-04-20

**Authors:** Filip Mandys, Mona Chitnis, S. Ravi P. Silva

**Affiliations:** 1 School of Economics, University of Surrey, Guildford, UK; 2 Research & Market Analysis Division, European Investment Fund, Luxembourg, Luxembourg; 3 Research Institute for Labour and Social Affairs, Prague, Czech Republic; 4 Surrey Energy Economics Centre, School of Economics, University of Surrey, Guildford, UK; 5 Advanced Technology Institute, University of Surrey, Guildford, UK

**Keywords:** solar energy, renewable energy, solar photovoltaic, levelized cost of electricity, LCOE, solar electricity cost, grid parity

## Abstract

Solar photovoltaic (PV) electricity represents one of the most promising sources of clean and affordable energy; however, the share of solar power in electricity production remains low, primarily because of the high installation costs. By conducting a large-scale analysis of electricity pricing, we show that solar PV systems are quickly becoming one of the most competitive sources of electricity. Collecting a contemporary UK dataset of 2010–2021, we analyze the historical levelized cost of electricity for several PV system sizes, project until 2035, and conduct a sensitivity analysis. The cost of PV electricity is currently at about 149 ₤/MWh for the smallest-scale and 51 ₤/MWh for large-scale PV systems, already lower than the wholesale price of electricity, with PV systems predicted to get cheaper by 40%–50% until 2035. The government should focus on supporting solar PV system developers with benefits such as simpler land purchases for PV farms or preferential loans with low interest rates.

## Highlights

• The cost of PV systems has been steadily falling in the last decade, regardless of system size
• The 2021 cost of electricity was 149 ₤/MWh for small and 51 ₤/MWh for large-scale PV systems
• Large PV systems in 2021 were cheaper than wholesale electricity; small PV systems will break even by 2027
• PV systems are predicted to get cheaper by 40%–50% by 2035

## The bigger picture

A growing number of countries worldwide are concerned about the adverse effects of anthropogenic climate change and the gradual depletion of fossil fuels. Many countries are therefore committed to finding ways of reducing their carbon emissions and ultimately reaching carbon neutrality. Solar photovoltaic (PV) electricity represents one of the most promising sources of clean and renewable energy, but it has suffered in the past from steep costs. Our research uses the latest available data to estimate past UK solar PV system costs while accounting for several key assumptions, and it projects the costs until 2035. This analysis can provide a better understanding of the expansion potential of solar PV systems, even in high-latitude countries. Lower PV system costs and the related potential for increased diffusion of this technology could make a huge difference in the overall carbon emissions of a society. Cost estimates of this kind may help governments and others make better decisions in the short and long term regarding PV system policies and investment.

## Graphical abstract

**Figure:**
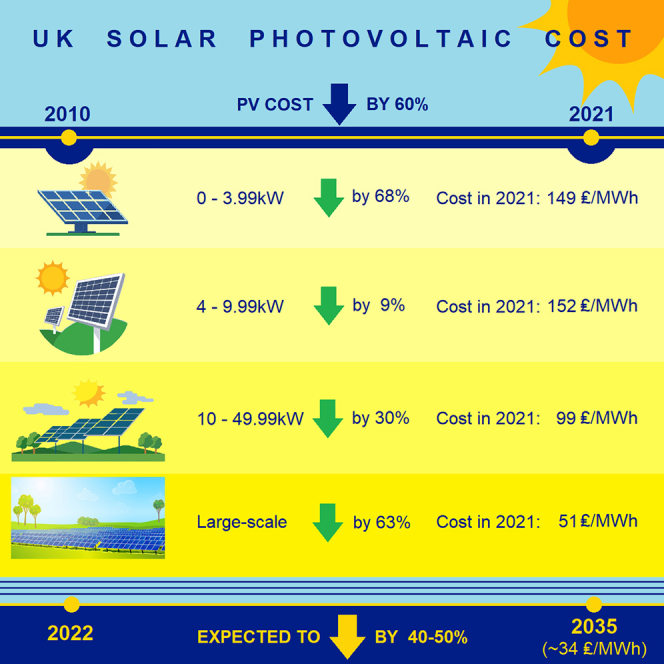

## Teaser

This research evaluates the past UK levelized cost of electricity for several PV system sizes and makes projections of the costs until 2035 using current data from 2010–2021. The findings confirm that the cost of solar PV systems has decreased substantially in the last decade, with solar PV electricity production getting cheaper with system size. Large-scale PV system cost is already lower than the wholesale electricity price and is predicted to get cheaper by 40%–50% by 2035, making solar PV systems very attractive.

## Introduction

Electricity lies at the heart of most current modern and green technologies, and therefore its global demand has increased significantly over time, with expectations for it to increase even more substantially in the future.^1^ Electricity is the most versatile form of energy provision and has the most potential for decarbonization worldwide. Hence, effective methods of how to generate electricity consistently, cheaply, and sustainably are currently of great interest to researchers, governments, developers, and the public around the world.

Despite the abundance of the solar resource in the world, the share of solar power in electricity production remains low at around 3% globally in 2019,^2^ primarily because of the very high installation costs. Nevertheless, this 3% share is the result of a rapid solar photovoltaic (PV) expansion over the last decade. This swift expansion is projected to continue into the future.^2^^,^^3^ However, the pace of change is not in line with the needs of reaching a net-zero world by 2050, and more needs to be done to show how the levelized cost of electricity (LCOE; the “discounted lifetime cost of building and operating a generation asset, expressed as a cost per unit of electricity generated;”^4^ it can be used to compare the costs of generating electricity from various power sources over time), even in countries within the northern hemisphere as far north as 55°, can have positive impacts with its adoption. The change in trajectory for countries close to the equator toward solar electricity has been rapid, with over 55% of new installations worldwide in large-scale programs adopting such a route. The reason for this growth and the positive predictions is primarily the cost decline of PV technology; since 2010, the cost of solar PV systems dropped by about 85%, and therefore, generating electricity from large-scale solar systems is now cheaper than coal or gas, even in the more northern countries.^5^

A recent editorial points to the fact that, over the last decade, LCOE future price predictions have notably underestimated the solar module prices because they have not taken into account the very-large-scale deployment happening worldwide.^5^ When price is corrected, solar PV system cost for the present and in the future is likely to make PV systems the cheapest new energy generation technology. This positive outlook and cost reductions can be further reinforced by implementation of new promising technologies, such as perovskite or quantum dot solar cells, which are cheap, flexible, stable, and efficient because of use of inorganic nanostructures.^6^ This trend has taken place since the introduction of fourth-generation PV systems in the early 2000s.^7^

There is no question that a mixed bag of energy supplies is needed to provide the energy needs of a nation. In the past, these have been totally dominated by fossil fuels but have more recently been supplied through nuclear, wind, and solar as clean routes of energy provision. In terms of the national electricity grid, clean energy provision makes up a majority of the generating sources at present, with wind becoming the dominant electricity provider, particularly in the north of the UK. However, based on careful macroeconomic cost models conducted by the UK government in terms of real cost data on 2018 prices, large-scale solar PV system generating costs have been shown to be lower than that of offshore or onshore wind.^4^^,^^8^ Furthermore, the cost of solar PV systems worldwide has been decreasing at a faster rate than the cost of wind, with cost estimates expected to further decrease with increased installed capacity.^5^ The cost of wind power is also becoming more competitive with time, and there is an overall greater installed wind capacity than solar PV in the UK and northern hemisphere countries, with a higher load factor.^9^ Nevertheless, in terms of true levelized cost estimates, large-scale solar PV deployment is still cheaper and will get a bigger boost with more energy storage capacity installed in the country.

For many countries, the government promise of cheap, clean, and renewable electricity generation on a large-scale is crucial because of the aforementioned projected rise in electricity demand (this projected rise in electricity demand is also contributed to by various sectors that aim to become cleaner as well; for example, a switch from conventional to electric vehicles will be only partly effective in terms of reducing emissions if most of the extra electricity generated would come from fossil fuels), the rising concerns about climate change, the gradual depletion of fossil fuels, and government targets of net-zero emissions by 2050. Similarly, because of rising consumer electricity prices, knowledge of small-scale PV system costing and projections is increasingly more important for consumer planning and decision-making regarding installation of rooftop solar PV panels. These reasons have attracted great interest of many parties in the costing dynamics, economic incentives, and trends of solar PV systems in different countries.

Economic incentives have a key role in investment decision-making for solar projects on any scale. Many countries have introduced support schemes to encourage investment in solar projects at different scales to overcome the barrier of high initial capital cost and make such projects profitable over time for the investors, given the electricity price in the market. While such support mechanisms are necessary when the initial capital cost is high, they impose a cost burden on the government or other authorities. These costs are normally passed on to final consumers in some way, which, in turn can reduce consumers’ economic welfare. For instance, the transfer of support cost burden contributing to consumer electricity price increase affects energy poverty. It is therefore important to understand the level of need for such economic support (if any) and expose potential investors to economic information about solar projects in relation to the electricity market. For example, the changes in the investment capital cost of solar energy over time as the technology improves (and significantly affects the decision about the level of support mechanisms) and information about cost effectiveness of solar projects can naturally attract more investors. On the other hand, expansion of solar electricity generation in a market based on merit order (economic) dispatch can reduce the price of electricity in the wholesale market because the marginal cost of solar electricity generation is zero (merit order effect, examined for various European countries by, e.g., Welisch et al.,^10^ Luňáčková et al.,^11^ and Wen et al.^12^). This reduction in wholesale electricity prices in the absence or reduction of economic support for solar projects can eventually transfer to electricity retail prices, helping to achieve the promise of clean and affordable electricity.

To advance our understanding of this, there is a need for a comparison of solar electricity generation costs and electricity prices in the appropriate market for various electricity generation scales. However, the literature on current and projected costings for solar energy is limited despite its importance for planning and investment decisions. To fill this gap in the literature and demonstrate the importance of such an analysis, in this study we focus on the UK as our case study; however, our analysis can easily be extended to other countries or regions, subject to data availability. Furthermore, it is clear that the farther south we go from the UK, the improved sunlight hours in the day will make the economic arguments so much stronger and compelling for transition to solar PV systems. Therefore, the analysis we conduct for the UK can be considered a worst-case scenario in terms of lower sunlight hours, high labor costs for installation of such large-scale programs, and competition from other efficient renewables, such as wind power.

In particular, the originality of this study lies in examining the solar PV system costing dynamics using a newly constructed and contemporary dataset for the UK from 2010–2021, collected from a wide range of sources, and applying the LCOE approach with different assumptions to calculate the historical unit cost of PV electricity for various system sizes (while we take into account disaggregated costs of solar PV systems, such as development and operation and maintenance costs, we do not include external costs, such as the carbon footprint of manufacturing PV technologies, cost to human health, loss of biodiversity, etc.). Furthermore, we project the UK LCOE for various PV system sizes until 2035 using different scenarios and assumptions and conduct a sensitivity analysis for an extensive combination of key variables. Such an empirical analysis provides a significant update to the current academic literature because, although a general LCOE analysis has been conducted before, contemporary LCOE evaluations of the UK and detailed future projections have been limited despite their importance for planning and investment decisions. Thus, to the best of our knowledge, previous studies have not applied the LCOE to an extensive dataset, providing accurate current and projected costings. To achieve the national targets of net-zero emissions, the government needs to be clear in what the trends and developments of solar PV systems are, whether the costs are decreasing and at what rate, and how quickly this technology is expected to get cheaper in the future. To achieve the net-zero emissions, there have been changes in many areas of the UK, in particular in the transportation sector with the diffusion of electric vehicles (e.g., Mandys^13^), the industry sector with introduction of carbon taxes (e.g., Martin et al.^14^), the households sector with introduction of green energy programs (e.g., MacPherson and Lange,^15^ Taneja, and Mandys^16^), or even academic institutions (e.g., O’Flynn et al.^17^). Therefore, the results of this study will provide a better understanding of the UK PV system costing dynamics, allow better planning of PV capacity increase by the UK government, and allow evaluation of whether we may have access to nearly free electricity within the next two decades.

The rest of the paper is organized as follows. Solar energy in the UK explains the UK’s solar energy situation. Literature review reviews the relevant solar energy literature. Data sources covers the sources and collection of the data used in the analysis. Experimental procedures discusses the LCOE approaches, the method used for future projections, and the sensitivity analysis. Results presents the results of the study. Conclusion concludes the paper.

### Solar energy in the UK

There are two main methods of harnessing solar energy to convert into electrical energy: solar PV systems and concentrated solar power (CSP) systems. CSP systems are more suited to arid and semi-arid areas, while PV systems are more appropriate for middle to high latitudes, particularly in locations with partly cloudy weather.^18^ Because the focus of this study is on the UK (which gets its solar energy from PV systems), only solar PV systems will be considered in this paper. PV electricity represents one of the most promising sources of renewable energy in the UK, reaching cost parity with onshore wind despite the UK government’s preferential treatment of the latter technology.^8^^,^^19^ Rising demand for electricity and the UK government goal of net-zero emissions by 2050 has attracted great interest of many parties in the costing dynamics and trends of solar PV systems.

**Figure 1:**
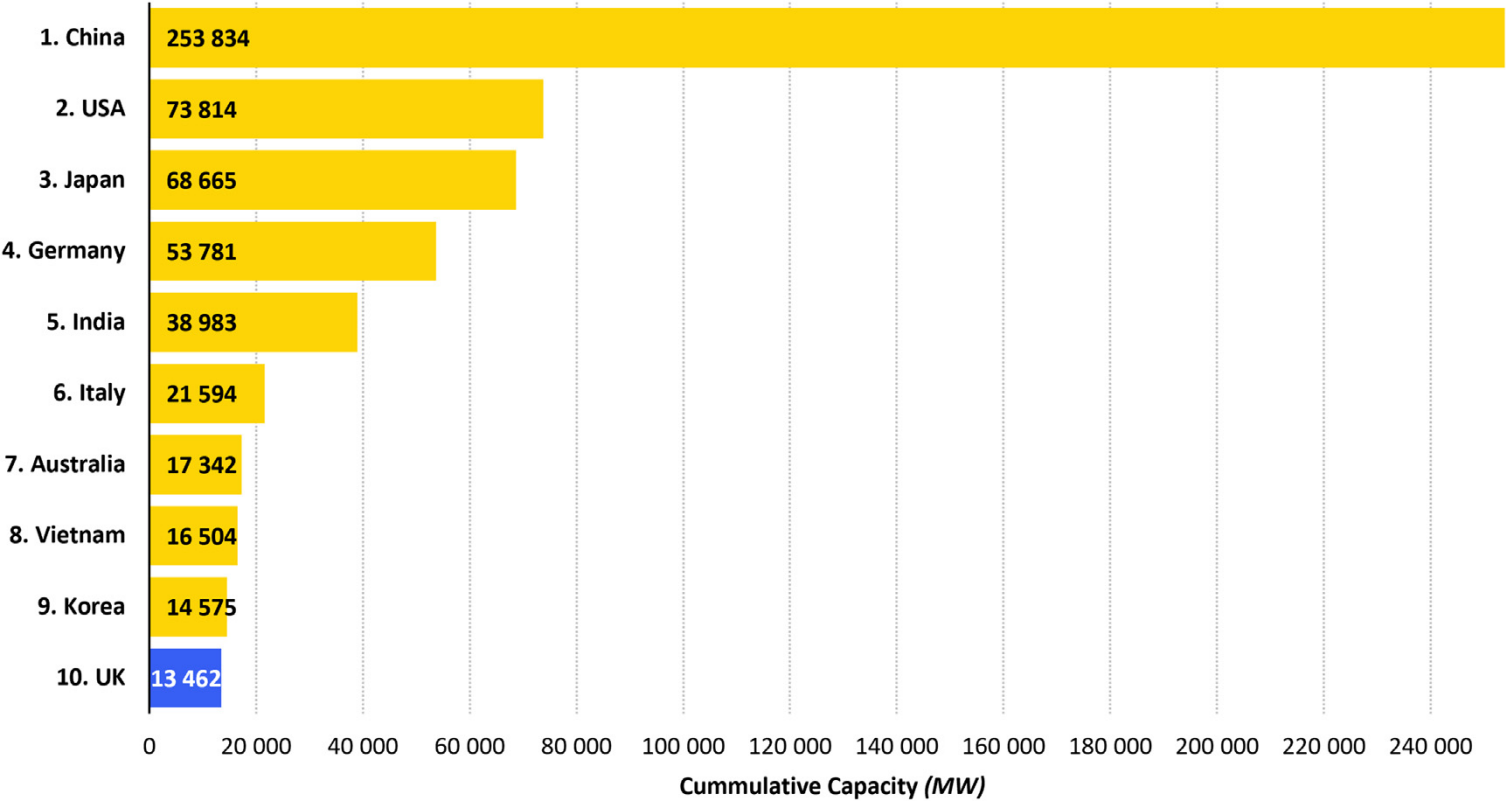
Cumulative capacity of solar PV systems by country in 2020, measured in megawatts (taken from IRENA)^21^

The trend of PV system development in the UK has been associated with quick expansion of the cumulative capacity and the number of installations. The first larger-scale systems came online during 2009, with a total capacity of only 0.03 GW, but this quickly rose to almost 13.5 GW in 2020, with about 12.5 TWh of electricity generated.^20^ In 2020, Cleve Hill in Kent, UK, obtained planning permission to set up a subsidy-free 350-MW solar farm, which will act as a showcase for the establishment of PV systems in the UK and countries far from the equator. This establishes the UK as the country with the third-largest PV capacity in Europe and 10th in the world (Figure 1). The increase in the UK’s capacity is projected to continue in the near future, reaching circa 21 GW in 2025 and around 29 GW in 2030.^22^

**Figure 2:**
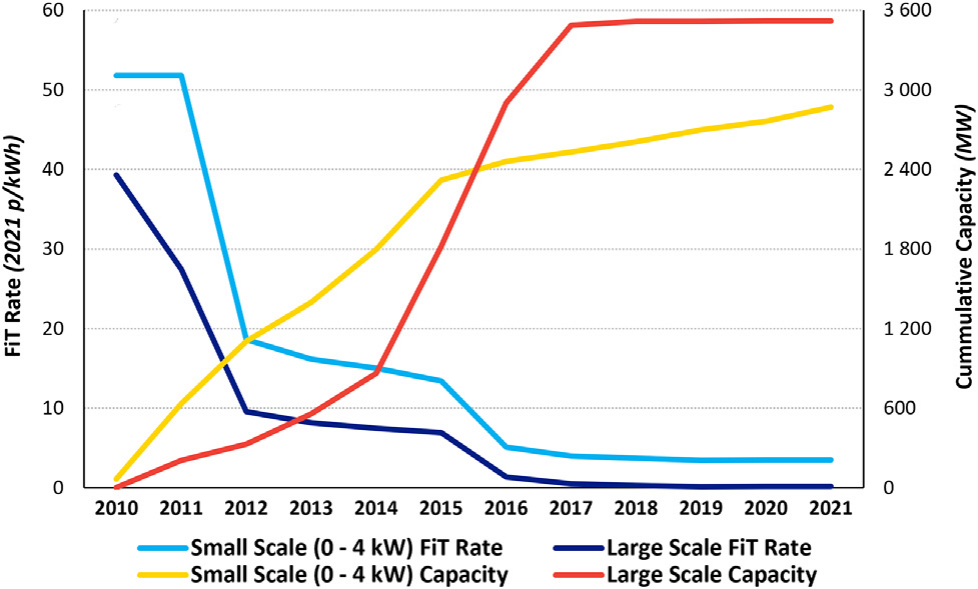
Cumulative capacity of PV systems in the UK of various sizes and the corresponding FiTs rates (2010–2021, taken from BEIS and OFGEM)

The high adoption rates of solar PV systems in high-latitude countries, such as the UK, may seem counterintuitive at first, and some may argue that they are unnecessarily expensive in relation to the limited sunlight. However, as Ondraczek et al.^23^ point out: “the factors influencing the cost of solar PV … include more than just the level of sunshine.” Their results suggest that northern countries may have lower PV LCOE and thus should have indeed subsidized solar PV technologies. The idea that solar PV systems are, in fact, competitive in countries such as the UK even without subsidies is supported by the UK market data, which shows that, despite the large decrease in the PV feed-in tariff (FiT) rates during the last several years, the PV cumulative capacity at each plant size continued to grow (Figure 2). This trend has also been illustrated recently with the 2020 approval of the Cleve Hill Solar Park, the UK’s largest (350 MW) projected solar park, which will be unsubsidized.

A swift increase in PV capacity induced by cost reductions of the PV technology is an imperative goal for the UK government, especially in relation to the promised reductions of greenhouse gas emissions and the country’s target of net-zero emissions by 2050. In relation to that, the UK plans to gradually replace the conventional vehicle fleet with electric vehicles, which further requires substantial amounts of extra electricity generation. According to the Committee on Climate Change (CCC),^24^ all new cars and vans should be electric by 2035 at the latest. The predicted net costs of switching to electric cars are lower for a 2030 conventional vehicle phase-out compared with a more gradual 2040 phase-out.^24^ This finding further stresses the importance of planning an increase in the cumulative capacity of UK PV systems in advance, based on the PV system costing dynamics. If the UK, and the world in general, does not expand its renewable energy generation with solar PV systems having a crucial role, it will be unable to satisfy the growing energy demand in the coming years^1^ or curb the harmful emissions causing environmental change.^24^

## Literature review

Previous literature on the solar energy sector focuses primarily on the aspects and costing methodologies of solar PV. These studies mainly employ some form of the life cycle costing (LCC; a process of measuring all of the costs that will be spent on an asset over the course of its lifetime) or LCOE methodologies. However, other studies with relevance to our paper are also explored. This includes other renewable energies (predominantly wind or CSP), novel assumptions, and alternative methodologies (e.g., levelized cost of storage).

### LCOE methods

One of the most popular methods of analyzing the costing aspects of various energy sources is the LCOE methodology. Consequently, this method is frequently used to address the costs of renewable energies, such as solar PV systems. One of the earlier works in this area is the paper by Branker et al.,^25^ attempting to review the LCOE of solar PV systems as well as clarify the most important assumptions of the method. Using a 2009 dataset for Canada, the authors make several recommendations on how to correctly normalize an LCOE analysis while controlling key variables, such as the project system costs, lifetime, and loan term. Similarly, Darling et al.^26^ focused on explanation of the underlying assumptions of the LCOE calculations. One of the key recommendations is to report a range (or distribution) of the LCOE values rather than a single calculated value. An increasing number of researchers focus on the LCOE of solar energy in China because the country currently has the largest (and rapidly expanding) cumulative capacity in the world. Authors point to the need of greater FiTs for renewable energies, combined with greater subsidies, to promote the growth of renewable power sources.^27^ Nevertheless, even without generous subsidies, renewable power sources, such as CSP, have increasingly competitive LCOEs in China^28^ as well as other countries.^29^ Even other, less traditional solar power sources are becoming increasingly more competitive. A study by Mulligan et al.^30^ using Australian data showed that organic solar PV systems could become competitive in the foreseeable future, even when compared with conventional power sources, such as coal or oil. At 2% efficiency and a 3-year lifetime, organic PV systems would be competitive with conventional PV, while at 5% efficiency and a 4-year lifetime, they would compete even with coal-based electricity generation. With these predictions in mind, it may be profitable for many consumers to install their own solar PV panels in the foreseeable future and at least partially rely on their own electricity production. According to Mundada et al.,^31^ if the US with 2014 costs is considered, then the technological developments and economies of scale of solar PV panels, batteries, and combined heat and power systems bring the possibility for a considerable number of consumers to indeed leave the grid (grid defection). However, it is important to note that batteries for solar PV energy storage systems are not in an advanced state as yet, and they do come with several problems. These include their relatively high cost, suitability only for a short-term variability in energy production, and limited number of charging cycles. Apart from the established countries (such as China or the US), solar PV systems may also have a surprising potential in northern countries.^23^ Calculating a global LCOE for over 140 countries, the authors find that the LCOE of solar PV systems in high-latitude countries may, in fact, be lower than in equatorial countries because of varying financing costs, and therefore subsidizing solar PV systems in these countries may be a promising strategy. A similar finding of economic benefit in high-latitude countries was reported for Canada by Robertson et al.^32^

The LCOE methodology is not limited to calculations of the historical costs but it can also be applied to project the costing in the future. Hernández-Moro and Martinez-Duart^18^ used data from Germany, Spain, California, and Northern Africa to predict the development of solar PV and CSP up to 2050. The simulation of the LCOE suggested that the cost reduction will be faster for CSP in the first several years, but over time it would become slower than large-scale deployment of solar PV systems. Therefore, solar PV systems would be most appropriate for mid to high latitudes, while CSP technology would best fit arid areas. A similar approach was taken by Parrado et al.,^33^ predicting the LCOE of solar PV and CSP until the year 2050 in Chile.

Aspects such as intermittency or environmental concerns are not accounted for by the standard LCOE methodology; however, several studies aimed to expand it and include these additional aspects. Reichelstein and Sahoo^34^ adapted the LCOE method to include a co-variation coefficient, a value that accounts for intermittency when interacting with LCOE. Using 2012 US data, the authors discovered that such an adjusted LCOE for solar PV systems is 10%–15% lower compared with standard LCOE. An alternative way of accounting for intermittency was explored by Hirth et al.^35^ using their system LCOE. Their method can control for value and cost differences, which mainly involve intermittency and variability of solar PV electricity generation. Other factors affecting the variability of solar PV systems were explored by Marzo et al.^36^ Using data from Chile, the authors extended the LCOE framework to also consider the effect of “atmospheric extinction” (the effect where direct solar irradiation is partially extinguished in its path between the mirrors of a CSP plant [heliostats] and the receiver tower^36^) on solar irradiation. Similar to Reichelstein and Sahoo,^34^ Sinha et al.^37^ used 2012 US data to account for environmental and performance aspects in the LCOE calculations. Even when controlling for these factors, PV power systems were found to be competitive with conventional power generation. Several authors attempted to include the costs of storing generated electricity in their LCOE calculations. Pawel^38^ created a model for combining solar PV LCOE and storage LCOE. Such a combined model could arguably be useful for costing calculations in power plants that combine solar PV electricity generation and storage. Storage LCOE was also analyzed more recently by Lai and McCulloch,^39^ who additionally developed a levelized cost of delivery (LCOD) method, the LCOE for electrical energy storage. Applying the LCOD, the authors concluded that Li-ion batteries would be sub-optimal for electrical energy storage and that using vanadium redox flow batteries would be preferable. A recent study in the UK by Durmaz et al.^40^ examined the effect of smart meters, applying the data of Ridley et al.^41^ Using their new method of levelized cost of consumed electricity (LCOCE), the authors show that accounting for smart meters reduces the cost of electricity compared with the standard LCOE procedure.

### Other methods

Apart from the LCOE method and the various expansions, many authors analyze the costing aspects of renewable energies using the LCC technique (this is a process of measuring all of the costs that will be associated with an asset over the course of its lifetime). The method was examined in more detail by Laura and Vicente,^42^ using Spanish data for illustration. The authors identified six distinct phases of LCC, specifically for several types of offshore wind platforms. Such an LCC analysis can allow evaluation of module, operation, and maintenance costs in new power plants, as in Abu-Rumman et al.^43^ However, the LCC method is frequently considered insufficient for full costing analysis unless it is integrated into a complete sustainability assessment model. Because the LCOE technique intrinsically depends on LCC calculation (Equation 1), using LCC alone may often be sub-optimal without factoring in environment, economy, and social equity.^44^ Such an analysis was attempted for Spain by Corona et al.,^45^ using their full environment LCC (FeLCC) method. The results suggested that the highest costs in power plant lifetime come from construction (48%) and operation and maintenance (23%), while disposal costs are only negligible.

Other approaches of analyzing solar PV systems includes the merit order effect. The merit order effect was calculated, for example, for Denmark, Germany, and Spain;^10^ the Czech Republic;^11^ and New Zealand.^12^ Regardless of the approach taken and the renewable energy analyzed, however, the authors found a negative effect of higher renewable energy penetration on the wholesale market price of electricity. An exception is the solar PV technology in the Czech Republic, which is found to have a positive effect on wholesale electricity prices.^11^ Apart from the methods discussed, many authors choose more unique and original methods for the costing analysis. Such methods include various implementations of software analysis, such as RetScreen by Rehman et al.,^46^ or systematic mapping and survey analysis, as in Benes and Augustin.^47^

## Data sources

### Research data

The dataset used for the analysis in this study is collected from a wide range of sources and has information on many energy-related variables primarily for the UK. Additionally, for comparison purposes, data on several other countries are also included, along with averages for the whole world. The primary sources that were used for data collection include the UK Department for Business, Energy, and Industrial Strategy (BEIS); the International Renewable Energy Agency (IRENA); the Office of Gas and Electricity Markets (OFGEM), the European Photovoltaic Industry Association (EPIA); Solar Energy UK; the CCC; and the Office for National Statistics (ONS). Because these are official as well as governmental data sources, the data collected can safely be considered reliable and of high quality. Several secondary sources were also used for missing or singular additional variables, such as the World Bank, or the BP (formaly the British Petroleum company) reports. All of the collected data and variables from the different sources were combined into a complete dataset covering the time period from 2010–2021. Because the UK only had negligible solar PV electricity production before this time period (and, consequently, lack of data), the time period from 2010 was chosen for analysis.

The key variables collected are the cumulative capacity of the solar PV systems installed (disaggregated by the size of the PV systems) and the disaggregated cost of the solar PV systems in the UK (including, e.g., development costs, module costs, operation and maintenance costs, etc.). These data are crucial for the different proposed LCOE calculations and projections that represent the core part of this study. The cumulative capacity data come from the official Solar Photovoltaics Deployment in the UK database of the BEIS.^48^ The database includes the total installed UK cumulative capacity for each month, disaggregated across different sizes of the solar PV systems. The annual cumulative capacity data are taken as the capacity at the end of each particular year (i.e., in December of each year). The cost of the PV systems, on the other hand, is a difficult variable to obtain because the total fully disaggregated costs are rarely publicly available, and the available data usually represent only a rough estimate and are frequently not available on a year-to-year basis. Therefore, our data come from several different sources and are disaggregated into small-scale PV systems (0–3.99 kW, 4–9.99 kW, and 10–49.99 kW) and a large-scale PV system (over 50 kW). These specific system capacities are selected to correspond to the system capacities of the BEIS, which provides the data for the system costs and the cumulative capacity. The data for the small-scale PV systems costs come from the official Annual Cost of Small-Scale Solar Technology Summary database.^49^ This data source includes information on separate costs of different aspects of small-scale solar PV panel installation as well as overall average costs. The data for the large-scale PV system costs come from the BEIS,^4^ Solar Energy UK,^21^ and Lugo-Laguna et al.^50^ This includes disaggregated costs across several variables, such as the costs of development (design, financing, etc.), operation and maintenance, insurance, business rates, modules, inverters, or grid connection.

Other variables that were collected from the official and governmental data sources include information on the installations that participated in the FiT scheme (rates received, disaggregated by installation size), the total electricity generation from solar PV systems, the solar PV load factors (a load [or capacity] factor represents the average effectiveness of an installation, calculated by comparing the installation’s real generation output with the potential maximum output; for the world, the PV average is around 18%, while for the UK, this is lower, at around 11%.), wholesale annual electricity prices, and historical values of large-scale PV LCOE for Germany, Spain, the US, and China, as a comparison with the UK. The official data for solar PV electricity generation and solar PV load factors are taken from IRENA,^2^ BP,^3^ and the UK Renewable Electricity Capacity and Generation database.^51^ This includes the annual amount of electricity that was generated solely from solar PV systems and the annual average UK solar PV load factors in percentage terms. The information about the FiT system and rates is taken from the official data of OFGEM.^52^ The data for the UK wholesale electricity prices is taken from OFGEM^53^ Wholesale Market Indicators on an annual basis. The information on the historical LCOE of large-scale solar PV systems of different countries comes from the official report of IRENA.^54^

The descriptive raw data values of some of the key variables used in the analysis can be seen in Table 1. As can be seen in the table, the cumulative capacity of solar PV systems across different system sizes has increased considerably between 2010 and 2021. This is especially the case for large-scale systems, where capacity increased over 3,000-fold, from only 3 MW to almost 9,500 MW. At the same time, average total PV costs have been consistently falling over the time period examined, with the smallest system size average cost falling by almost 70% since 2010. The UK solar PV load factor has been fairly consistent since 2014, at around 11%. Additionally, wholesale electricity prices have been gradually falling between 2010 and 2020, with a sharp increase following in 2021, increasing wholesale electricity prices almost 3-fold compared with 2020.

**Table 1:** Descriptive raw data values of several key variables (2010–2021)

Variable (2010–2015)	2010	2011	2012	2013	2014	2015
Cumulative capacity, 0–3.99 kW (MW)	65	636	1,104	1,397	1,797	2,320
Cumulative capacity, 4–9.99 kW (MW)	6	43	73	106	139	179
Cumulative capacity, 10–49.99 kW (MW)	6	96	235	337	461	651
Cumulative capacity, large scale (MW)	3	208	343	1,034	3,058	6,481
Load factor (%)	7.5	5.1	11.2	9.8	10.9	11.4
Wholesale electricity price (₤/MWh)	55.33	57.51	52.06	57.11	46.79	44.62
Avg. total PV cost, 0–3.99 kW (₤/kW)	5,210	3,607	2,670	2,336	2,317	2,066
Avg. total PV cost, 4–9.99 kW (₤/kW)	–	–	–	1,864	1,789	1,628
Avg. total PV cost, 10–49.99 kW (₤/kW)	–	–	–	1,585	1,538	1,420

Variable (2016–2021)	2016	2017	2018	2019	2020	2021
Cumulative capacity, 0–3.99 kW (MW)	2,460	2,531	2,607	2,699	2,764	2,870
Cumulative capacity, 4–9.99 kW (MW)	199	215	236	268	297	348
Cumulative capacity, 10–49.99 kW (MW)	722	764	809	876	910	952
Cumulative capacity, large scale (MW)	8,403	9,166	9,323	9,405	9,448	9,448
Load factor (%)	11.0	10.6	11.2	10.7	10.9	10.1
Wholesale electricity price (₤/MWh)	46.45	49.58	60.67	44.68	44.11	114.67
Avg. total PV cost, 0–3.99 kW (₤/kW)	2,06	1,966	1,891	1,688	1,617	1,666
Avg. total PV cost, 4–9.99 kW (₤/kW)	1,645	1,585	1,577	1,634	1,693	1,701
Avg. total PV cost, 10–49.99 kW (₤/kW)	1,353	1,244	1,190	1,110	1,082	1,104

The prices and costs are deflated, in 2021 Pounds Sterling. Avg., average.

## Methodology

### Historical LCOE

To achieve the main goals of this study, we calculate the historical costs of solar PV electricity as well as its future projections. First, the approach involves application of the LCOE approach with different assumptions for the UK between 2010 and 2021. Second, we apply an additional method to project the UK LCOE for the period of 2022–2035 using the predicted UK future solar PV cumulative capacity and the PV learning rate. Additionally, we perform a sensitivity analysis by accounting for all different combinations of key variables that affect the LCOE calculations in all years examined. This includes the discount rate, project lifetime, learning rate (the percentage reduction in costs for each doubling of cumulative capacity), load factor, degradation rate, and escalation rate (the expected percentage annual increase of the operation and maintenance costs). The LCOE is calculated and projected for four different PV system sizes: small scales of 0–3.99 kW, 4–9.99 kW, and 10–49.99 kW and large scales (50 kW and above).

The LCOE methodology is an established, accepted, and widely used technique (see, e.g., Ouyang and Lin,^27^ Mundada et al.,^31^ and Durmaz et al.^40^) for estimating the cost and effectiveness of producing power from a certain technology. The method is frequently implemented in academic papers and in official reports of organizations, such as the International Energy Agency (IEA) or IRENA. The general assumptions used for calculating the LCOE are summarized, for example, in Branker et al.;^25^ however, LCOE can typically be defined as the ratio between the discounted total lifetime cost of the project (life cycle cost) and the discounted total lifetime energy generated (life cycle energy production):

(Equation 1) $$LCOE=\frac{Life\;Cycle\;Cost}{Life\;Cycle\;Energy\;Production}$$

Therefore, the LCOE calculates a net present value, measured, for example, in pounds per megawatt hour. Therefore, LCOE can be defined as the price at which electricity needs to be sold to break even over the lifetime of the project;^26^ thus, lower costs generally imply greater competitiveness of the technology.

For this study, we apply the LCOE calculation with two different set of assumptions for comparison purposes as well as LCOE projections (Table 2). Both sets of assumptions account for various values of the discount rate, project lifetime, and load factor; however, each set also has additional specific variables for which it controls. The first set of assumptions, inspired by, e.g., Darling et al.^26^ and Mundada et al.,^31^ accounts additionally for the degradation rate of the PV systems, as presented in Equation 2:

(Equation 2) $$LCOE=\frac{I+\Sigma_{t=1}^{T}\frac{OM}{{\left(1+r\right)}^{t}}+\Sigma_{t=1}^{T}\frac{LP}{{\left(1+r\right)}^{t}}-\Sigma_{t=1}^{T}\frac{SV}{{\left(1+r\right)}^{t}}}{\Sigma_{t=1}^{T}\frac{h\cdot f{\left(1-d\right)}^{t}}{{\left(1+r\right)}^{t}}}$$

where *I* is the initial capital cost (₤/MW), *OM* is the yearly operational and maintenance cost (₤/MW), *LP* is the yearly loan repayment cost (₤/MW), *SV* is the scrap value at the end of the project (₤/MW), *h* is the average number of hours in a year, *f* is the load factor, *d* is the degradation rate, *r* is the discount rate, and *T* is the project lifetime.

**Table 2:** Summary of key variables controlled for in different assumption sets

Variable	Historical LCOE		Projected LCOE
	Assumption set 1	Assumption set 2	Assumption set
	(Equation 2)	(Equations 3 and 4)	(Equation 10)
Learning rate (ρ)	–	–	yes
Discount rate (*r*)	yes	yes	yes
Project lifetime (*T*)	yes	yes	yes
Load factor (*f*)	yes	yes	yes
Degradation rate (*d*)	yes	no	yes
Escalation rate (*e*)	no	yes	no

Shown are key variables controlled for in different assumption sets when calculating historical and projected UK LCOE.

Because the initial capital costs *I* are paid at the project start (period 0), they need not to be discounted. If the scrap value *SV* is negative (i.e., adding to the cost of the project), then it would represent a decommissioning cost. Additionally, the number of hours in a year (*h*) is calculated as 24 (hours in a day) × 365.25 (days in a year) to account for the leap years during the lifetime of the project, and interacted with the UK load factor (*f*).

The second set of assumptions for the LCOE calculations, used, for example, in Narbel et al.,^55^ does not control for the degradation rate of the PV system (i.e., d=0), but does additionally account for the escalation rate, as presented in Equation 3:

(Equation 3) $$LCOE=\left[\frac{\left(\frac{r{\left(1+r\right)}^{T}}{{\left(1+r\right)}^{T}-1}\right)\cdot I}{h\cdot f}\right]+\left[L\cdot \frac{OM}{h\cdot f}\right]$$

where

(Equation 4) $$L=\left(\frac{r{\left(1+r\right)}^{T}}{{\left(1+r\right)}^{T}-1}\right)\cdot \left(\frac{1+e}{r-e}\right)\cdot \left(1-{\left(\frac{1+e}{1+r}\right)}^{T}\right)$$

In the above equations, *e* is the escalation rate and *L* is the levelization factor. The levelization factor *L* integrates the increase of the operation and maintenance costs as the plant ages. While Equations 2 and 3 differ in their control for different assumptions and variables, such as the escalation rate, levelization factor, and degradation rate, their respective resulting LCOE values tend to be fairly comparable, even when performing sensitivity analysis (using all possible combinations of all key variables). This fact brings further robustness and confidence to our results and to our subsequent conclusions.

### LCOE projections

For the calculation of the UK LCOE projection from 2022–2035, we use a modified version of the LCOE methodology. This method attempts to project the future evolution of LCOE for a certain PV system size based on the technology learning rate and historical values, and the future planned UK cumulative capacity (the UK historical values are for the last 10–12 years, dependent on system size, because earlier, the UK had only negligible production of electricity from solar PV systems and virtually no PV farms). When applying the LCOE projection approach, we follow the method of Hernández-Moro and Martinez-Duart,^18^ adopted, for example, by Zhao et al.^28^ If we represent the yearly costs of a solar PV plant as a constant percentage of the initial costs of the PV system and modify our Equation 2 accordingly, then the LCOE would take the form

(Equation 5) $$LCOE=\frac{I+\Sigma_{t=1}^{T}\frac{I\cdot \left(OM_{p}+LP_{p}\right)}{{\left(1+r\right)}^{t}}}{\Sigma_{t=1}^{T}\frac{h\cdot f{\left(1-d\right)}^{t}}{{\left(1+r\right)}^{t}}}$$

where $OM_{p}$ and $LP_{p}$ represent the constant percentage of yearly costs (e.g., operation and maintenance and loan repayment) of the initial costs *I*. As mentioned above, historical LCOEs calculated using Equations 2 and 3 are similar. Therefore, we only use the modified Equation 2 for calculating the projected LCOE.

**Figure 3:**
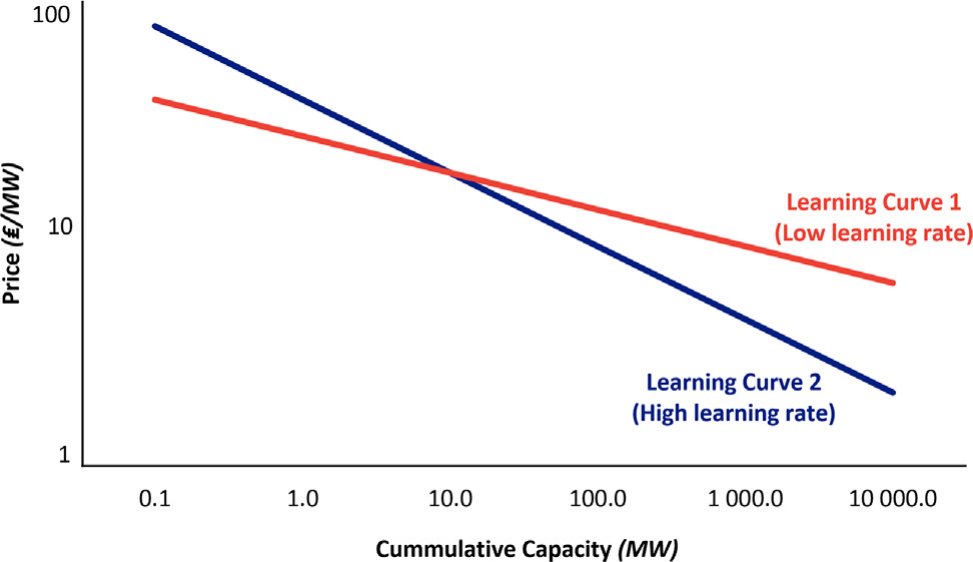
Example of learning curves with different learning rates The higher the learning rate, the faster the price of the technology decreases for every increase in cumulative capacity.

As noted by the IEA,^56^ electricity production from renewable power sources tends to decline according to economies of scale. For every increase in cumulative capacity, there is a reduction in production costs, and this decrease can be characterized using a learning curve. An example of two distinct learning curves with different levels of learning rate is shown in Figure 3.

Following Figure 3, one can calculate the learning rate of a technology by examining the evolution of the costs of the technology (e.g., balance of systems costs) for every doubling of cumulative capacity and use it to project this evolution into the future.^57^ As noted by IRENA,^54^ “on average, in 2019, balance of system costs (excluding the module and inverter) made up about 64% of total installed costs.” Following Hernández-Moro and Martinez-Duart,^18^ as learning curves are straight lines in a log-log space, their equation at times $t_{1}$ and $t_{2}$ may be written as

(Equation 6) $$\log\left(I_{t_{2}}\right)=-b\left(\log\left(Q_{t_{2}}\right)-\log\left(Q_{t_{1}}\right)\right)+\log\left(I_{t_{1}}\right)$$

which can be rewritten as

(Equation 7) $$\frac{I_{t_{2}}}{I_{t_{1}}}={\left(\frac{Q_{t_{2}}}{Q_{t_{1}}}\right)}^{-b}$$

where *Q* is the cumulative capacity at different points in time *t*, and *b* is the slope of the learning curve (straight line in a log-log space).

Because *b* is the slope of the learning curve, and the learning rate represents the percentage decrease of the cost for every doubling of the cumulative capacity, their relationship can be summarized as

(Equation 8) $$1-\rho =2^{-b}-b=\frac{\log\left(1-\rho \right)}{\log\left(2\right)}$$

where ρ is the learning rate. Consequently, when we use Equation 8 and implement it into Equation 7, we find that the cost of the PV system *I* at some future point of time *t* is given by

(Equation 9) $$I_{t}=I_{0}{\left(\frac{Q_{t}}{Q_{0}}\right)}^{\frac{\log\left(1-\rho \right)}{\log\left(2\right)}}$$

where $I_{0}$ and $Q_{0}$ are the system costs and cumulative capacity at the initial time 0. Therefore, when we modify Equation 5 for the LCOE calculation using Equation 9, we get an expression for calculating the LCOE at any time period in the future, as in, e.g., Hernández-Moro and Martinez-Duart,^18^ Parrado et al.,^33^ or Zhao et al.^28^:

(Equation 10) $$LCOE_{t}=\frac{I_{0}{\left(\frac{Q_{t}}{Q_{0}}\right)}^{\frac{\log\left(1-\rho \right)}{\log\left(2\right)}}+\Sigma_{n=1}^{N}\frac{I_{0}{\left(\frac{Q_{t}}{Q_{0}}\right)}^{\frac{\log\left(1-\rho \right)}{\log\left(2\right)}}\cdot \left(OM_{p}+LP_{p}\right)}{{\left(1+r\right)}^{n}}}{\Sigma_{n=1}^{N}\frac{h\cdot f{\left(1-d\right)}^{n}}{{\left(1+r\right)}^{n}}}$$

where *N* is the project lifetime.

This means that we can calculate the future LCOE at any point in time based on the initial capital costs $I_{0}$, initial cumulative capacity $Q_{0}$, the future predicted country cumulative capacity $Q_{t}$, and the learning rate ρ, assuming a stable discount rate through the lifetime of the project.

Finally, when performing the sensitivity analysis for both historical LCOE approaches and the LCOE projection, we control for all possible levels and combinations of the key variables, calculating the effect on costs of each variable in turn. Table 3 shows the assumptions used for the key variables, where the low value represents the lowest possible value the variable can take, and the high value represents the highest possible value.

**Table 3:** Values of key variables used for the LCOE calculations

Variable	Low value	Middle value	High value	Source
Learning rate (ρ)	32%	37%	42%	IRENA
Discount rate (r)	3.5%	7.5%	10.0%	BEIS
Project lifetime (T)	25 years	30 years	35 years	BEIS
Load factor (f)	9%	11%	13%	IRENA, BEIS
Degradation rate (d)	0.10%	0.39%	0.80%	Solar Energy UK
Escalation rate (e)	0.5%	2.0%	5.0%	BEIS, Bank of England

Shown are values of key variables used in calculating the UK historical and projected LCOE. The different values are taken from the reports and data of the IRENA, BEIS, and Solar Energy UK.

## Results

**Figure 4:**
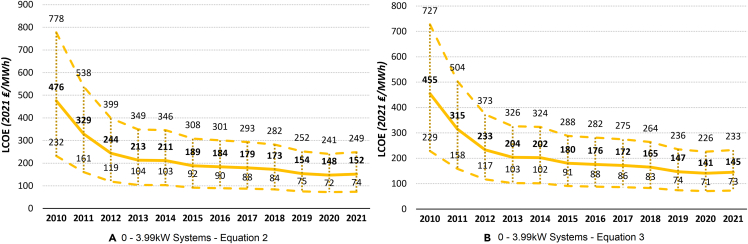
The UK historical LCOE of 0 to 3.99 kW system size over time The thick middle line represents the mean LCOE, while the upper and lower dashed lines represent the maximum and minimum estimated LCOE, respectively. (A) 0 to 3.99 kW systems (Equation 2). (B) 0 to 3.99 kW systems (Equation 3).

**Figure 5:**
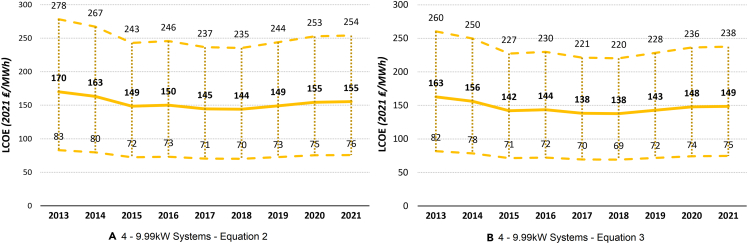
The UK historical LCOE of 4 to 9.99 kW system size over time The thick middle line represents the mean LCOE, while the upper and lower dashed lines represent the maximum and minimum estimated LCOE, respectively. (A) 4 to 9.99 kW systems (Equation 2). (B) 4 to 9.99 kW systems (Equation 3).

**Figure 6:**
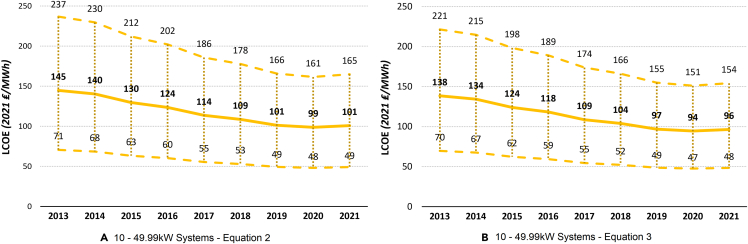
The UK historical LCOE of 10 to 49.99 kW system size over time The thick middle line represents the mean LCOE, while the upper and lower dashed lines represent the maximum and minimum estimated LCOE, respectively. (A) 10- 49.99 kW systems (Equation 2). (B) 10- 49.99 kW systems (Equation 3).

**Figure 7:**
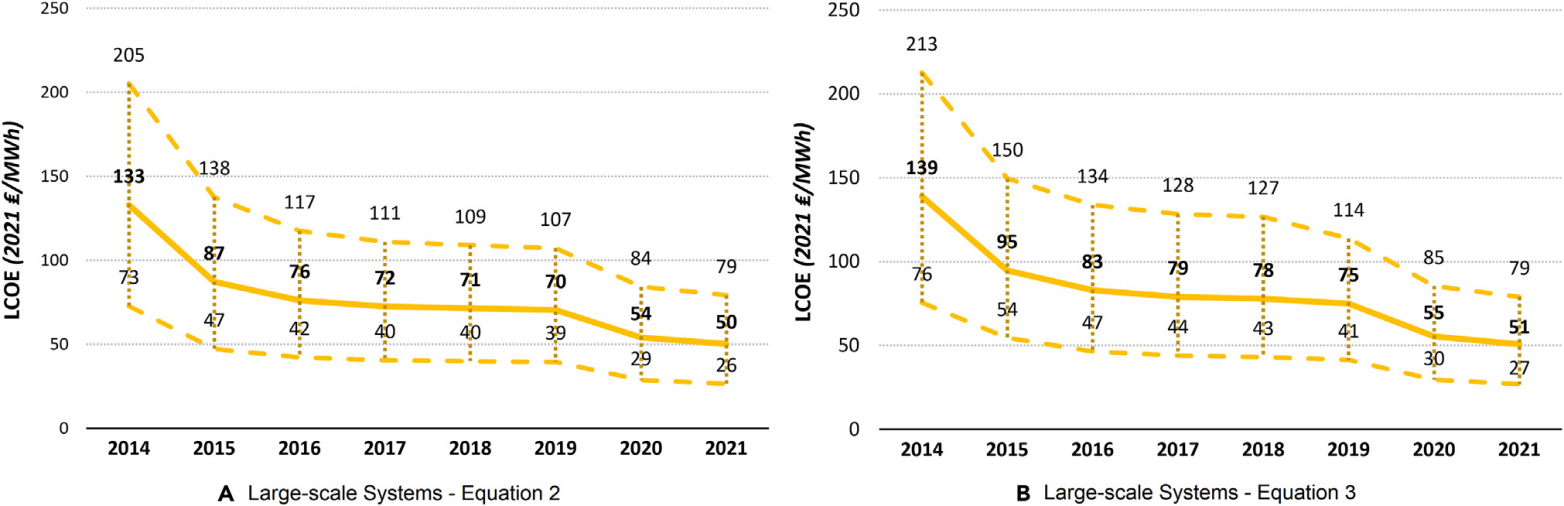
The UK historical LCOE of large-scale system size over time The thick middle line represents the mean LCOE, while the upper and lower dashed lines represent the maximum and minimum estimated LCOE, respectively. (A) Large-scale systems (Equation 2). (B) Large-scale systems (Equation 3).

Both sets of the LCOE assumptions in Equations 2 and 3 account for various levels of the discount rate, project lifetime, and load factor. However, Equation 2 additionally accounts for different levels of system degradation rate, while Equation 3 accounts for the escalation rate (the expected percentage annual increase of the operation and maintenance costs). The LCOE results for four different PV system sizes and the sensitivity analysis curves can be seen in Figures 4, 5, 6, and 7.

### Historical UK LCOE

From our results below, it is clear that the cost of UK solar PV electricity is quickly decreasing over time, across all PV system sizes for both approaches used. Although the cost decrease is slowing down over time, it is still very significant, even in the last several years. For example, the large-scale PV system cost decreased by a significant 26 ₤/MWh (30 €/MWh (Equation 2; the transfer from 2021 Pounds Sterling to 2021 Euros was calculated using the average 2021 exchange rate; according to the European Central Bank, the average exchange rate in 2021 was ₤1 = €1.1634^58^) and 32 ₤/MWh (37 €/MWh) (Equation 3) over the last 5 years. This represents a very promising outlook for the future adoption rates and support of solar PV systems by the UK government, especially with this trend expected to continue in the future.

Comparing Equations 2 and 3, we get very similar results across the board, showing robustness of our findings to different assumptions and variable values. Equation 2, on average, estimates slightly higher LCOE compared with Equation 3 for all PV systems, except for large-scale PV systems. In general, the larger the system size, the less costly electricity generation is, consistently through time. While in 2021, the middle LCOE for 0- to 3.99-kW systems was 152 ₤/MWh (177 €/MWh) (Equation 2) and 145 ₤/MWh (169 €/MWh) (Equation 3), for large-scale PV systems, this was only 50 ₤/MWh (58 €/MWh) and 51 ₤/MWh (59 €/MWh), respectively. This means that large-scale solar PV system is, at the moment, the most competitive PV system size in terms of electricity generation cost. Because of its simplicity, renewable nature, and continuously decreasing costs, solar PV should be at the forefront of the government’s plans to greatly expand renewable electricity generation and reduce the carbon footprint of the UK.

**Figure 8:**
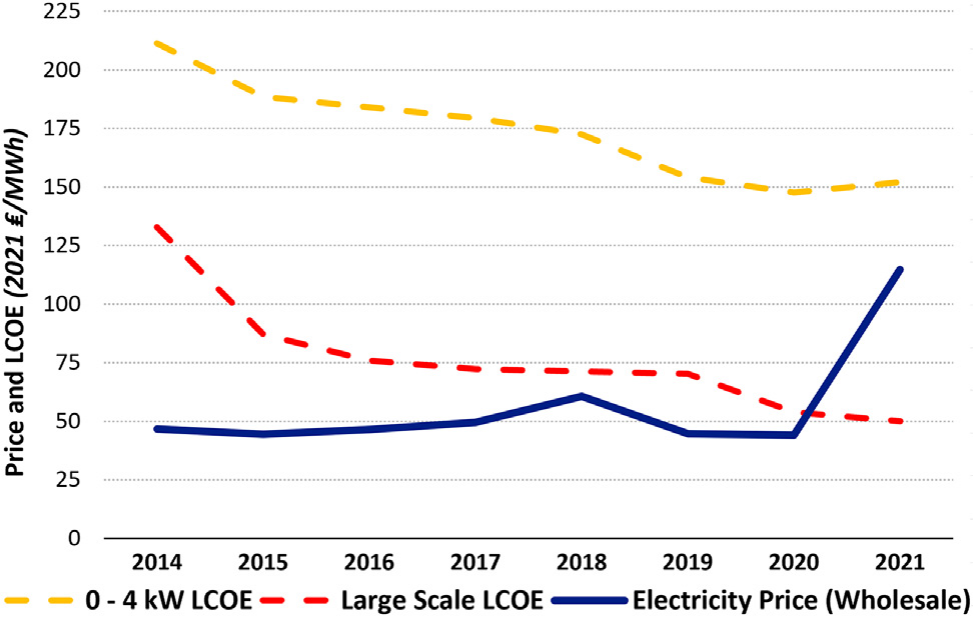
The increase of wholesale electricity and the decrease of small-scale and large-scale UK LCOE over time (taken from OFGEM)

While the smallest PV system size (0–3.99 kW) is the most expensive of the four sizes compared, increasingly greater numbers of households and firms are expected to acquire these; e.g., as roof or wall mounts. Because of the decreasing costs of PV systems and the rising costs of electricity across all sectors, in particular during the current energy crisis in Europe, small size PV systems are becoming increasingly more attractive for domestic and commercial use (despite the significant reductions in FiT rates; Figure 2). The increase in wholesale electricity prices and the reduction in costs of small- and large-scale PV systems are shown in Figure 8.

The trend of the last 8 years shows that large-scale PV systems became cheaper than the wholesale electricity price in 2020 because of a decrease in solar PV costs and sharp increase in electricity prices. Nevertheless, even the smallest-scale PV system (0–3.99 kW) cost is quickly approaching the wholesale electricity price and thus becoming increasingly more attractive. In 2021, the cost of electricity of a 0- to 3.99-kW system was only about 35 ₤/MWh (41 €/MWh) away from the price of wholesale electricity, considerable progress since 165 ₤/MWh (192 €/MWh) in 2014. With this trend continuing, it is possible that it will soon be cheaper for households to generate at least part of their own electricity from roof- or wall-mounted solar PV panels than to purchase electricity from the grid. This argument, following from our analysis, can give even a more dynamic reason for change when retail electricity pricing is considered alongside wholesale electricity pricing. Therefore, in a matter of a decade, we may see that a sizable proportion of UK domestic electricity is produced using clean and renewable solar PV energy. This would be especially likely during the summer months, when solar power is the least limited by its seasonal variability.

**Figure 9:**
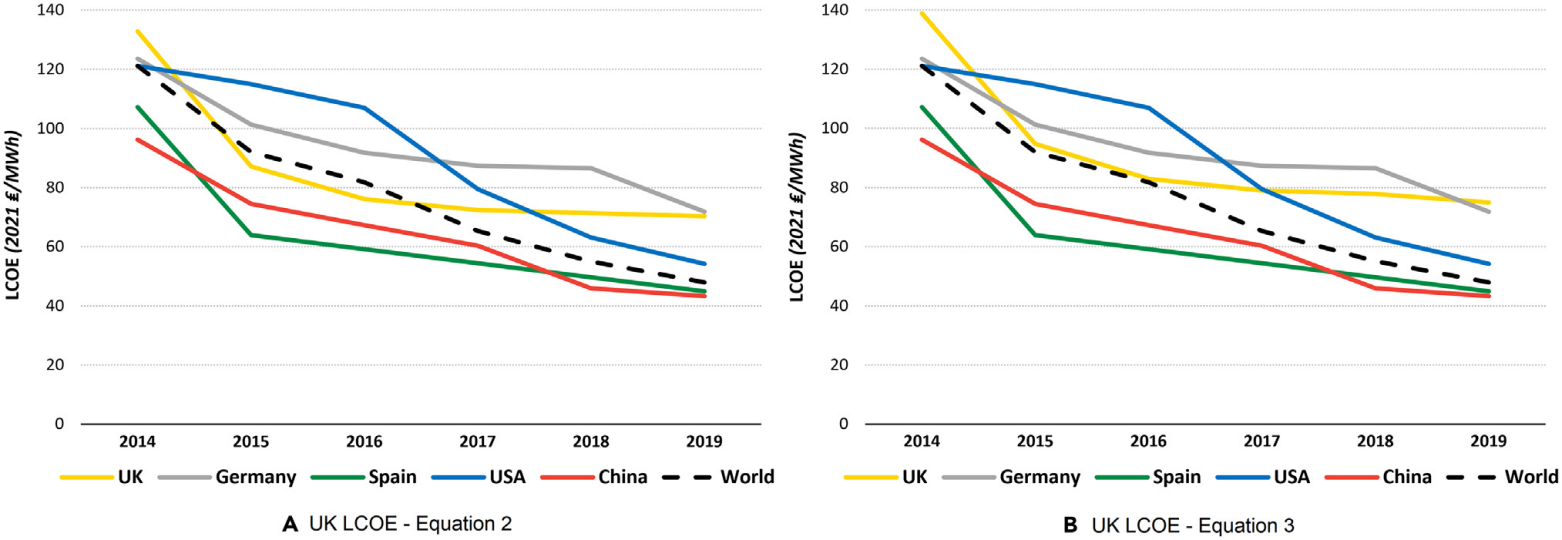
Comparison of the UK LCOE with various large-scale solar PV-generating countries (taken from IRENA) (A) UK LCOE (Equation 2). (B) UK LCOE (Equation 3).

As the UK cost of PV electricity has been significantly decreasing for the last decade, we compared the UK LCOE with several other leading countries of solar PV systems for international comparison (Figure 9). Spain was chosen because it has one of the lowest LCOEs, the US and China are world leaders in solar PV capacity, and Germany’s development closely matches the one of the UK. The high latitude of the UK means that the solar PV electricity cost is consistently higher than in lower latitude countries such as Spain and faces greater seasonal variability of production. On the other hand, because of their large size, countries such as the US and China span multiple climate zones and solar radiation levels. Therefore, the LCOE for the USA and China decreased faster than in the UK over the past years. In terms of cost development, the UK is quite similar to Germany, especially when Equation 3 of our LCOE estimation is applied. Nevertheless, despite the higher UK LCOE compared with countries like Spain, solar PV systems in the UK are becoming increasingly more competitive, and because of the government’s goals of emission reductions, the scale and cumulative capacity are expected to quickly rise in the near future.^20^^,^^21^

### Projection of UK LCOE

**Figure 10:**
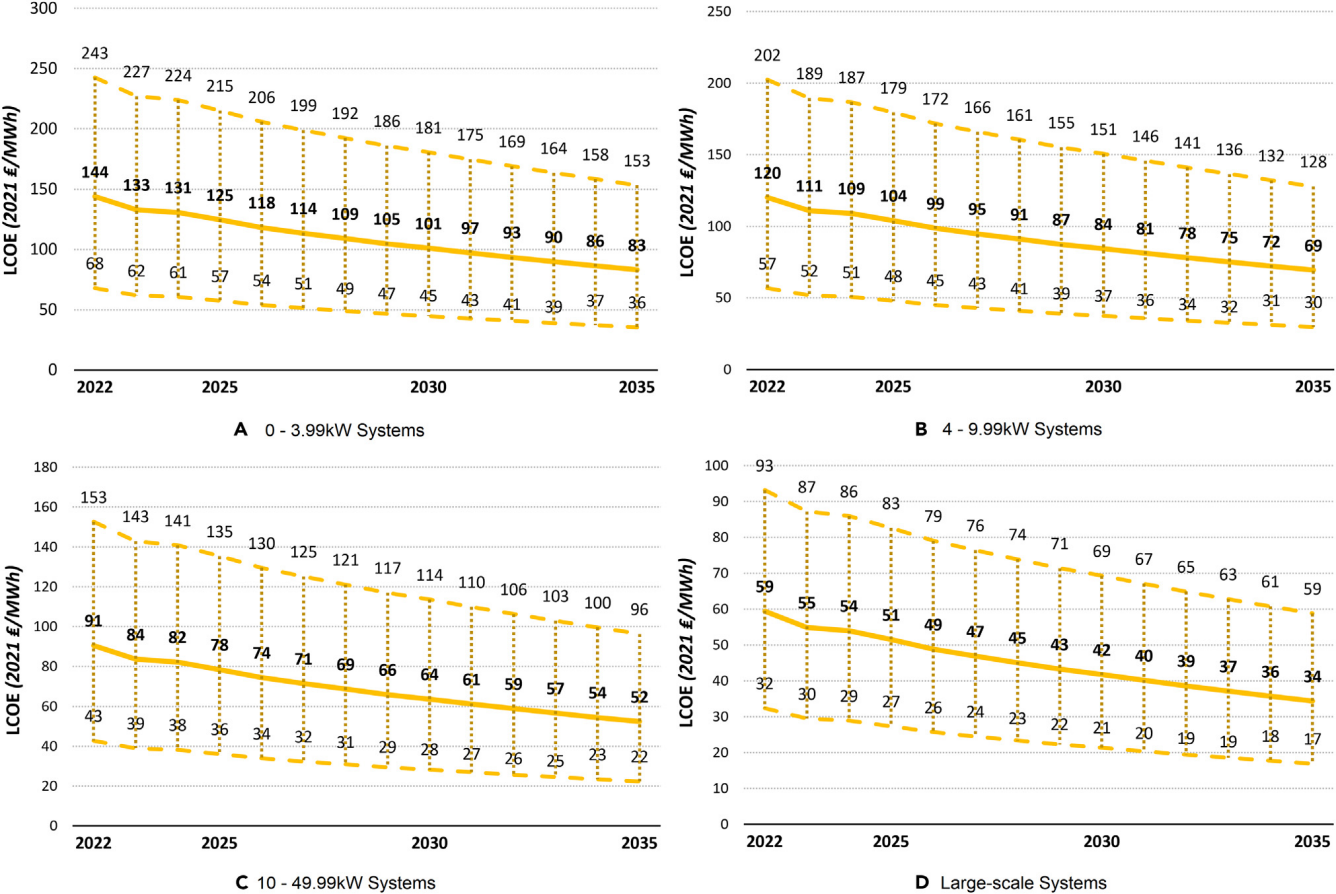
The projected development of the UK LCOE for PV system sizes during the period of 2022–2035 The thick middle line represents the mean LCOE, while the upper and lower dashed lines represent the maximum and minimum estimated LCOE, respectively. (A) 0 to 3.99 kW systems. (B) 4 to 9.99 kW systems. (C) 10 to 49.99 kW systems. (D) Large-scale systems.

To provide a more tangible expectation of the cost dynamics and UK LCOE development in the future, we calculate the UK LCOE projections from 2022–2035 for the four different PV system sizes considered. These projections are based on the UK cumulative capacity history and predictions as well as the expected learning rate (the percentage reduction in costs for each doubling of cumulative capacity) for solar PV systems. Similar to the historical LCOE calculations above, a sensitivity analysis is performed for the projections, controlling for all possible combinations of the key variables: discount rate, project lifetime, degradation rate, and load factor. Additionally, because of the importance of the learning rate for the subsequent results, sensitivity analysis is also performed for this variable. Robust solar PV LCOE projections for different system sizes can be very useful for all involved parties, such as the UK government, solar developers, and individual households. Knowledge of the likely path the solar PV system cost will take in the future is crucial for effective planning and accurate investment decisions with respect to the evolving energy markets. The calculated projections for the four PV system sizes are shown in Figure 10.

Our results indicate that the UK LCOE is predicted to significantly decrease by 2035, regardless of the PV system size considered. Similar to the historical LCOE, the projections suggest that the future LCOE will continue decreasing over time, at a slower rate. As shown in Figure 10D, while large-scale PV systems are expected to decrease by almost 3 ₤/MWh (3.50 €/MWh) per year in terms of cost until 2025, this will reduce to about 1.50 ₤/MWh (1.75 €/MWh) per year in terms of cost between 2030 and 2035. Therefore, considering the sensitivity analysis for all system sizes, the cost of solar PV systems is expected to decrease by 40%–50% over the next 15 years. This means that large-scale solar PV systems are likely to become increasingly more competitive to the point of becoming one of the cheapest ways to generate electricity on a large scale, even cheaper than onshore and offshore wind energy, which are predicted to cost 44 ₤/MWh (51 €/MWh) and 43 ₤/MWh (50 €/MWh), respectively, in 2035.^4^ These results, however, should be approached with caution because the raw cost of the technology shows only one side of the story. Despite the low cost, electricity production from solar PV systems would still face a number of challenges in the form of intermittency and seasonal variability in generation, the need for large-scale energy storage capacity, and good overall connections to the grid. Compared with onshore and offshore wind power, solar PV systems would also fall behind in terms of efficiency because wind turbines are currently able to harness a greater percentage of energy passing through them. Nevertheless, the efficiency of PV panels is continuously increasing, and our results suggest that, in the most optimistic scenario, the LCOE of large-scale UK solar PV systems would plummet to only 17 ₤/MWh (20 €/MWh), significantly below the costs of other electricity-generating technologies. Similar are the projections for small PV system sizes, suggesting that the cost of generating electricity from the 0- to 3.99-kW system will be lower than the price of wholesale electricity around the year 2027 (this may be even sooner if the price of wholesale electricity continues to rise). Therefore, it can be expected that many households and businesses would want to install even small-scale solar PV panels to reduce their electricity costs and promote environmental consciousness. Therefore, our findings suggest that, to speed up the fall of solar PV system costs, promote cheap and clean energy, and consequently achieve the target of net-zero emissions, the UK government should enhance their subsidy and support programs for solar PV systems to speed up the expansion of solar PV systems and increase the share of this technology in electricity production for the next decade.

## Discussion and Conclusions

This study evaluates the progress of solar PV systems in the UK, focusing on the recent past and future costing dynamics and trends in electricity generation and cumulative capacity. This kind of information is crucial to many parties, such as governments, solar developers, investors, as well as the public in general, because of the forecasted rise in electricity demand, phasing out of fossil fuels, and the aim of the UK to reduce emissions and consequently reach net-zero emissions by 2050. The possibility of completely renewable, clean, and cheap electricity is of major interest to the UK government for their environmental targets. Similarly, because domestic electricity prices are continuously rising, understanding small-scale PV system costing and projections is increasingly more important for consumer decision-making regarding installation of rooftop solar PV panels. Therefore, the contemporary trends and future developments of PV system costs must be clear for effective planning and investment decisions. Because such contemporary costing evaluations and future projections have been limited in the past literature for the UK, we deliver a better understanding of the historical and future costing dynamics of solar PV systems by using a different set of assumptions for the LCOE analysis, applied to a contemporary dataset.

Our dataset was collected from a wide range of sources on the UK energy market in general and the PV system market in particular. This allowed for application of the LCOE methodology with two sets of assumptions, calculating historical PV electricity cost for four PV system sizes between 2010 and 2021. Furthermore, we used the LCOE methodology to project the development of these UK PV electricity costs until 2035, using predicted UK cumulative capacity and learning rates. Finally, we performed a sensitivity analysis on our results, considering all possible combinations of our key variables: discount rate, learning rate, load factor, project lifetime, degradation rate, and escalation rate.

Our results portray that the LCOE of solar PV systems in the UK has decreased significantly in the last decade and became significantly more competitive. Electricity production from solar PV systems gets cheaper with PV system size, where large-scale PV systems had an estimated middle LCOE of 51 ₤/MWh (59 €/MWh) in 2021 without government support, lower than the current wholesale price of electricity and other renewable technologies, such as wind power. While solar PV systems do face issues, such as the need for large energy storage systems, seasonal variability, and lower efficiency than, e.g., wind power, even the smallest PV systems are getting very price competitive, with the 0- to 3.99-kW system being only 35 ₤/MWh (41 €/MWh) away from the wholesale price of electricity in 2021. This suggests that small-scale PV systems are likely to become very attractive for household and commercial use in the near future, with the cost of electricity from the smallest system expected to match wholesale electricity prices by 2027. Furthermore, while the LCOE of UK PV systems is not yet as low as in countries such as Spain and faces greater seasonal variability, our projections suggest that the systems will get cheaper by 40%–50% by 2035, making solar PV systems an attractive mode of future power generation.

Consequently, the UK government should focus on supporting developers and investors, especially at early stages, with benefits such as simpler purchases of land for PV farms or preferential loans with low interest rates to speed up PV system development. Enhancing the government subsidy and support programs for solar PV systems would speed up development of large-scale solar farms, increasing the share of this technology in electricity production sooner, thus bringing a faster and smoother transition into clean, carbon neutral future. Therefore, although there is still a long way to go before solar PV systems become a permanent source of nearly free electricity for humankind, our analysis and results imply that the technology is overall on a bright path toward acquiring this life-changing status in the foreseeable future.

As a final note, there are a few limitations to our study that are out of the scope of this paper, which would allow possible future expansion of our research; for example, exploring the effect of additional external costs on PV LCOE, such as the cost to human health, loss of biodiversity, damage to crops and materials, or climate change, as in Corona et al.^45^ Similarly, while it is out of the scope of this paper to account for the effects of solar PV system seasonal variability and intermittency, examining these, as in, e.g., Reichelstein and Sahoo,^34^ could provide interesting additional insights into the UK renewables market. Similarly, potential expansion can account for additional aspects of solar PV systems, such as energy storage solutions to alleviate the problems of seasonal variation, like in Lai and McCulloch^39^ or Durmaz et al.^40^ Furthermore, performing a merit order analysis (see, e.g., Luňáčková et al.^11^ and Wen et al.^12^) for UK solar PV systems could also be of significant use for effective planning of PV developers and the UK government.

## Experimental procedures

### Resource availability

#### Lead contact

Further information and requests for data should be directed to and will be fulfilled by the lead contact, S. Ravi P. Silva (s.silva@surrey.ac.uk).

#### Materials availability

This study did not generate new unique materials.

## Data Availability

• The Excel dataset has been deposited online at Mendeley Data: https://doi.org/10.17632/58hf6ms3wj.1 and is publicly available as of the date of publication.^59^ • This paper does not report original code. The LCOE calculations are included in the deposited Excel dataset. • Any additional information required to reanalyze the data reported in this paper is available from the lead contact upon request.
